# Evaluation of Acute Locoregional Toxicity in Patients with Breast Cancer Treated with Adjuvant Radiotherapy in Combination with Pazopanib

**DOI:** 10.5402/2012/896202

**Published:** 2012-12-06

**Authors:** Sharad Goyal, Sneha Shah, Atif J. Khan, Hasan Danish, Bruce G. Haffty

**Affiliations:** Department of Radiation Oncology, The Cancer Institute of New Jersey, Robert Wood Johnson Medical School, UMDNJ 195 Little Albany Street, New Brunswick, NJ 08903, USA

## Abstract

*Purpose.* The purpose of this study was to analyze acute locoregional toxicity in patients with breast cancer receiving concurrent pazopanib and RT. *Materials and Methods.* Patients with breast cancer who received pazopanib in combination with radiation were identified and matched (2 : 1) to patients with breast cancer who did not receive pazopanib by use of chemotherapy, radiation field design, and radiation dose. Toxicity was scored by the Common Terminology Criteria for Adverse Events and statistical analysis was performed. *Results.* Grade 1 or 2 radiation dermatitis was seen in 100% and 84% of pazopanib and RT patients and matched controls respectively (*P* = NS). None of the patients receiving pazopanib and RT experienced ≥ grade 3 toxicity within the irradiated volume; three (16%) matched patients experienced a grade 3 skin reaction (*P* = 0.05). Interestingly, grade 1 or 2 hyperpigmentation was seen in 17% of pazopanib and RT patients and 60% of matched controls (*P* = 0.005). *Conclusion.* The addition of concurrent pazopanib and RT when treating the intact breast, chest wall, and associated nodal regions in breast cancer seems to be safe and well tolerated.

## 1. Introduction

The response of a tumor to ionizing radiation is dependent on several factors both intrinsic and extrinsic to the cancer cells. Intrinsic mechanisms of radioresistance include alteration of gene and protein expression resulting in selection of resistant variants [1]. The extrinsic tumor microenvironment varies anisotropically within a mass and is characterized by oxygen depletion, acidosis, glucose deprivation, and high lactate levels [2, 3]. Hypoxic cells are known to be resistant to the effects of radiation as oxygen is required to fix damage conferred by free radicals created by the ionizing radiation. Severely hypoxic cells can have an oxygen enhancement ratio of 2-3; this means they require 2-3 times the radiation dose of well-oxygenated cells for the same level of killing.

The tumor microenvironment is shaped by both the metabolic activity of cancer cells and circulation. For growth and survival, tumor cells and stroma secrete proangiogenic factors including FGF, PDGF, and the predominant factor VEGF, which result in endothelial cell migration and proliferation. This helps create new vessels often which are poorly functional with sluggish blood flow since VEGF causes vessel leakage and is expressed out of proportion to other angiogenic factors. This may result in persistent areas of hypoxia [1]. 

Therapies targeted at VEGF and other angiogenic factors are an active area of investigation. Recent studies show that antiangiogenic agents produce modest responses as single agents, but in combination with radiation and chemotherapy consistently improve tumor response [4]. Jain explains this seemingly paradoxical effect by proposing the concept of vascular normalization in which high VEGF levels produce poorly functional vessels impeding oxygenation and delivery of therapeutics [5]. Therefore, inhibition of VEGF improves vessel quality, enhancing delivery of chemotherapy and tumor oxygenation, resulting in increased radiation efficacy.

Pazopanib (GW786034 or Votrient, GlaxoSmithKline) is a second-generation, oral tyrosine kinase inhibitor (TKI) with multiple targets including VEGFR, PDGFR, c-kit, and FGFR. Pazopanib was recently FDA approved for the treatment of advanced renal cell carcinoma and soft tissue sarcoma. Clinical trials are ongoing in breast cancer, ovarian cancer, thyroid cancer, and cervical cancer. The most common adverse effects are nausea, hypertension, diarrhea, fatigue, vomiting, AST and ALT elevation, and hair color changes. Compared to other angiogenesis TKI, pazopanib shows a lower number of adverse effects that are of low grade when present [6]. This novel molecule has the potential to improve systemic disease and survival for many cancers with minimal toxicity compared to standard therapies. 

Literature on the safety of combination therapy involving Pazopanib and RT is lacking. The purpose of the present study was to perform an analysis of acute locoregional (within the irradiated volume) toxicity in patients with breast cancer treated with adjuvant combined modality therapy consisting of pazopanib and RT and to define this toxicity based on the Common Terminology Criteria for Adverse Events (CTCAE, v4.0).

## 2. Methods

After approval by the institutional review board, we identified patients with breast cancer treated with pazopanib through medical records. The acquired data were de-identified according to Health Insurance Portability and Accountability Act (HIPAA) guidelines. These patients were then cross-referenced with the radiation oncology database to determine if radiotherapy was delivered concurrently with respect to pazopanib; 12 patients were determined to have received concurrent treatment. Inclusion criteria included patients with a known diagnosis of breast cancer who were treated with concurrent pazopanib and radiotherapy. A group of control patients were identified and matched 2 : 1 based on age, use of chemotherapy, radiation field design, and radiation dose. Data including demographic variables, common comorbid conditions, surgery, concomitant chemotherapy, radiation dose and field design, cancer grade and stage, performance status, vital signs, and laboratory values were extracted.

Standard baseline evaluation included a complete medical history, physical examination, including performance status, and hematology, and clinical chemistry assessments. Patients were evaluated weekly during the course of radiotherapy, 3-4 weeks after completion of treatment, and then at 3–6 month intervals thereafter. To gather information regarding locoregional toxicities, charts were reviewed for presence of the following variables before, during, and after radiotherapy: fatigue, radiation dermatitis, skin hyperpigmentation, skin ulceration, soft tissue fibrosis, nausea, pneumonitis, diarrhea, hypertension, and anorexia. Toxicity was scored using the Common Terminology Criteria for Adverse Events (CTCAE, v4.0). In this system, sequelae are graded from mild (grade 1) to fatal (grade 5). Patients were considered to have a significant complication if they had a toxicity of grade 3 or higher. In cases in which a complication could have been the result of pazopanib and/or radiation toxicity, it was coded as radiation toxicity unless such symptoms predated the radiation treatment. 

Statistical analysis was performed using paired *t*-test for continuous variables and a chi-squared or Fisher exact test for nominal data when appropriate, with a *P* value of 0.05 or less indicating significance. A computer program package SAS (Version 9.1, SAS Institute, Cary, NC) was used for all statistical testing and management of the database.

## 3. Results

### 3.1. Patient Characteristics

Patients were identified to have received concurrently pazopanib and RT (*n* = 12) with a mean age of 46.8 years (range 31–59). All patients were stage III. There were 5 right-sided breasts and 7 left-sided breasts treated. Patients were treated to their intact breast after breast conserving surgery (*n* = 5) and to the chest wall after mastectomy (*n* = 7); of these, 11 patients received RT to the regional nodes. Matched patients were identified to have received radiation therapy alone (*n* = 25) with a mean age of 50.2 years (range 35–77). Patients were stage III (*n* = 24) or stage IV (*n* = 1). There were 9 right-sided breasts and 14 left-sided breasts treated. Patients were treated to their intact breast after breast conserving surgery (*n* = 6) and to the chest wall after mastectomy (*n* = 19); of these, 24 patients received RT to the regional nodes. See Table 1 for full details on patient characteristics.

### 3.2. Treatment Compliance

Preoperatively, pazopanib 800 mg PO daily began on Day 1 of the first paclitaxel cycle and continuing until 7 days before surgery. Postoperatively, pazopanib 800 mg PO daily began 4–6 weeks after surgery and continuing until 6 months after the first postoperative pazopanib dose. All patients were seen weekly during radiotherapy and at 1 or 3 months after completion.

All patients (100%) received concurrent pazopanib and RT without delay or treatment breaks during radiation therapy, and there were no treatment-related deaths. No patient (0%) experienced progression of disease while on therapy or received a treatment-related break secondary to adverse side-effects. After completion of radiation therapy, one patient stopped pazopanib a week early due to loss in weight. One patient chose to be taken off pazopanib 2 months after RT due to heightened nausea and fatigue. Two patients experienced elevated liver enzymes after being put on pazopanib and was thus taken off it. With a minimum followup of 6 months, 12 patients (100%) are alive and have completed therapy as planned.

### 3.3. Radiation Treatment Parameters

Opposed tangents directed at the intact breast to 5000 cGy followed by a tumor bed boost to a total dose of 6000 cGy in 200 cGy/day fractions were delivered to 11 (30%) patients; 10 of these patients received treatment to a supraclavicular (SCLV) field treated to 4600 cGy in 200 cGy/day fractions prescribed to a depth of 3 cm. Twenty-six (70%) patients received opposed tangents directed at the chest wall to 5000 cGy in 200 cGy/day followed by a tumor bed boost to a total dose of 6000 cGy in 200 cGy/day fractions (0.5 cm bolus was used on the chest wall for the initial 2000 cGy); a supraclavicular field treated to 4600 cGy in 200 cGy/day prescribed to a depth of 3 cm in 25 patients. For left-sided tumors, the amount of lung and heart shielded was up to the discretion of the treating physician. Patients were not treated using intensity modulation, respiratory gating, active-breath hold, or other such techniques. All patients were treated in the supine position. 

### 3.4. Toxicity

Grade 1 or 2 radiation dermatitis was seen in 100% and 84% of pazopanib and RT patients and matched controls, respectively (*P* = NS). None of the patients receiving pazopanib and RT experienced ≥ grade 3 toxicity within the irradiated volume; three (16%) matched patients experienced a grade 3 skin reaction (*P* = 0.05). Interestingly, Grade 1 or 2 hyperpigmentation was seen in 17% P-RT patients and 60% of matched controls (*P* = 0.005). 

Two patients (17%) experienced hair hypopigmentation while on pazopanib. One (8%) of these two patients also had skin hypopigmentation outside of the treatment field along with Grade 1-related hypertension. One other patient (8%) also experienced hair loss. One patient receiving concurrent pazopanib and RT developed a 1.5 cm area of ulceration (Grade 2) in the scar (central boost) at 3 months after completion of RT. She required local wound care and eventually received 40 hyperbaric oxygen treatments which completely resolved the ulceration. There was no other adverse locoregional toxicity attributable to the concurrent use of pazopanib and RT (Table 2). No patients receiving RT alone developed skin ulceration, pneumonitis, diarrhea, hypertension, nausea, anorexia, or liver abnormalities; one patient in the P-RT group developed grade 3 anorexia. 

In all patients, hemorrhage at or distant to the site of radiotherapy was not seen. Grade III–IV hematologic toxicities were seen in zero patients (0%) during the course of pazopanib and RT. Grade 1 or 2 fatigue was seen in 100% and 88% of P-RT patients and matched controls, respectively (*P* = NS). 

## 4. Discussion

In preclinical studies, vacsular endothelial growth factor inhibition has been shown to be both a chemosensitizer and a radiosensitizer [7]. A combination of antiangiogenic agents and radiation therapy may improve the therapeutic ratio by improving tumor kill while minimizing toxicities [8]. Pazopanib, an oral, angiogenesis inhibitor targeting VEGFR, PDGFR, and c-kit, has been studied in numerous types of tumors in the setting of clinical trials. At present, it has received FDA approval for the treatment of patients with advanced renal cell carcinoma and advanced soft tissue sarcoma (STS) who have received prior chemotherapy. The most common systemic side-effects associated with pazopanib have been diarrhea, hypertension, hair color change, nausea, fatigue, anorexia, and vomiting, alopecia, chest pain, dysgeusia, dyspepsia, and skin hypopigmentation.

Preclinical studies in immunocompromised mice indicated considerable dose-dependent tumor growth inhibition in xenografts such as colon, prostate, breast, renal, and lung [9]. Inhibition was most prominently seen in renal cell carcinoma xenografts, as 77% growth inhibition occurred with 10 mg/kg/day of pazopanib, with complete prevention of cell growth and multiplication at 100 mg/kg/day (3) [9]. Lung and colon xenograft growth was almost completely repressed at this dose. An important phase I trial tested 63 patients with advanced-stage and refractory solid tumors for increasing doses of pazopanib (3) [9]. A wide range of doses and schedules were used to evaluate optimal dosage and tolerability. Pazopanib was well tolerated by most patients, with most adverse events (AE) being of low-grade (1 or 2) and reversible (3) [9]. The most commonly reported drug-related AEs were hypertension, diarrhea, nausea, anorexia, fatigue, and hair hypopigmentation (3) [9]. The most common high-grade AE was hypertension, seen in 29% of patients (3) [9]. 

In the present study, the most common toxicities were fatigue and radiation dermatitis, both of which were related to the radiotherapy. In fact, there was a statistically lower rate of ≥  grade 3 dermatitis in patients receiving pazopanib compared to untreated patients. The addition of pazopanib to RT did not seem to increase the occurrence of acute or subacute locoregional toxicities compared to RT alone. Overall treatment was well tolerated, and no synergistic toxicities were seen. Interestingly, there was a statistically significant lower rate of hyperpigmentation in patients receiving pazopanib with RT compared to RT alone. A case report by Sideras et al. explored the underlying causes of skin and hair hypopigmentation in an African-American woman treated with pazopanib for thyroid cancer (4) [10, 11]. They postulated that given the role of c-Kit in melanocyte/pigmented cell proliferation and PDGF-R in melanocyte development, inhibition of c-Kit and PDGF-R by pazopanib would result in hypopigmentation. Radiation has been shown to modulate c-Kit/c-Kit ligand system in melanocytes causing an increase in pigmentation [12]. Thus it may be postulated that within the treatment field, the inhibitory effect of pazopanib on c-Kit outweighs any locoregional effect caused by RT on pigmentation.

The side effects caused by pazopanib that were observed in the present study were primarily systemic. These include fatigue, diarrhea, hypertension, nausea, and anorexia. When compared with the our matched patients, those receiving pazopanib reported a higher incidence of diarrhea, hypertension, nausea, and anorexia, but of low-grade toxicity. Fatigue occurred with roughly the same rate in both pazopanib and RT (33.3%) and the matched controls (31.6%). Only one incident of grade 3 toxicity (anorexia) was reported with pazopanib and RT, and that patient discontinued pazopanib therapy one week early. 

Vasudev and Larkin reported a phase III trial in which the most frequently reported AEs were diarrhea, hypertension, nausea, anorexia, and vomiting (5) [13]. The most common higher-grade toxicities (3/4) were hypertension (4%) and diarrhea (3%) (5) [13]. Recent studies may indicate that the frequency of certain toxicities increase as the plasma concentration of pazopanib goes up (5) [13]. This was demonstrated in data for diarrhea and hypertension, but was not seen for fatigue, nausea, or vomiting (5) [13]. This suggests that reducing dosages may be helpful in alleviating certain toxicities. Pazopanib has a lower incidence of fatigue than other TKIs, in part due to it also showing lower rates of thyroid problems when compared to other TKIs (5) [13]. However, pazopanib is also associated with hepatotoxicity as are other TKIs (5) [13]. 

Pazopanib has been approved for use by the U.S. Food and Drug Administration in the treatment of advanced renal cell carcinoma and soft tissue sarcoma. However, the U.S. FDA has issued warnings for pazopanib regarding its association with an increased risk of hepatotoxicity, QT interval prolongation, hemorrhagic events, arterial thrombotic events, hypertension, wound healing, hypothyroidism, and proteinuria. To our knowledge, data on the use of concurrent pazopanib and RT across tumor sites have not been reported in detail. However, pazopanib is being studied in phase II trials of anaplastic thyroid cancer in combination with paclitaxel and radiation therapy; in this trial, pazopanib is given concurrently with RT. Special attention must be given to those breast cancer patients with liver dysfunction, hypertension, and poor wound healing after surgery before starting targeted pazopanib therapy. Thus, it is necessary to fully evaluate the therapeutic ratio of antiangiogenic therapies used in combination with RT for safe use of this approach in the clinical setting. To our knowledge, this is the first study investigating acute locoregional toxicity in the adjuvant setting in breast cancer patients treated with concurrent pazopanib and RT to the intact breast or chest wall and associated nodal regions. 

Further research should explore and focus on efficacy and tolerability of VEGF inhibition concurrent with RT. Attention must be given to different modes of RT and appropriate pazopanib dosing and sequence studies when used in combination with RT. 

## 5. Conclusion

Our results show that when treating a patient's intact breast, chest wall, or associated nodal regions with RT in the adjuvant setting for breast cancer, concurrent pazopanib and RT do not increase acute locoregional toxicity as compared to matched control patients who receive similar RT treatment without use of pazopanib. With the increasing use of pazopanib, the addition of concurrent RT seems to be safe and well tolerated.

## Figures and Tables

**Table 1:** Patient characteristics.

Arm	Laterality	STAGE	CHEMO	Concurrent	SITE	RT Fields	RT DOSE
P-RT	R	III	AC-T + P	P	CW + Sclav	3	6000
P-RT	L	III	AC-T + P	P	CW + Sclav	3	6000
P-RT	L	III	AC-T + P	P	CW + Sclav	3	6000
P-RT	R	III	AC-T + P	P	Intact	2	6000
P-RT	R	III	AC-T + P	P	Intact + Sclav	3	6000
P-RT	R	III	AC-T + P	P	CW + Sclav	3	6000
P-RT	L	III	AC-T + P + H	P + Herceptin	Intact + Sclav + IM	3	6600
P-RT	R	III	AC-T + P	P	Intact + Sclav	3	6000
P-RT	L	III	AC-T + P + H	P + Herceptin	CW + Sclav	3	6000
P-RT	L	III	AC-T + P	P	CW + Sclav	3	6040
P-RT	L	III	AC-T + P	P	CW + Sclav and axilla	4	6000
P-RT	L	III	AC-T + P	P	CW + Sclav	3	6000
RT	R	IV	AC-T	//	CW + Sclav	3	6600
RT	L	III	AC-T + H	Herceptin	CW + Sclav	3	5800
RT	L	III	AC-T + H	Herceptin	CW + Sclav	3	6000
RT	R	III	AC-T	//	Intact + Sclav, IM, PAB/AAB	*5 *	6000
RT	R	III	AC-T	//	Intact + Sclav	3	6000
RT	L	III	TC, TAC	//	CW + Sclav	3	6000
RT	L	III	AC-T + H	Herceptin	CW + Sclav	3	6000
RT	L	III	TC + H	Herceptin	CW + Sclav	3	6000
RT	L	III	AC-T + H	Herceptin	CW + Sclav	3	6000
RT	L	III	//	//	CW	2	6600
RT	R	III	AC-T	//	Intact	3	6000
RT	L	III	TC + H	Herceptin	CW + Sclav	3	6000
RT	L	III	AC	//	CW + Sclav	3	6000
RT	L	III	AC-T	//	CW + Sclav	3	6000
RT	L	III	//	//	CW + Sclav	3	6000
RT	L	III	TC + H	Herceptin	CW + Sclav	3	6000
RT	R	III	//	//	Intact + Sclav	3	6000
RT	R	III	ECF	//	CW + Sclav	3	6000
RT	L	III	AC-T + H	Herceptin	CW + Sclav	3	6000
RT	R	III	AC-T	//	Intact + Sclav	3	6000
RT	R	III	AC-T	//	Intact + Sclav	3	6000
RT	R	III	TC, TAC	//	CW + Sclav	3	6000
RT	L	III	AC-T + H	Herceptin	CW + Sclav	3	6000
RT	L	III	TC + H	Herceptin	CW + Sclav	3	6000
RT	L	III	AC-T + H	Herceptin	CW + Sclav	3	6000

Abbreviations: P: pazopanib; RT: radiotherapy; L: left; R: right; A: adriamycin; C: cytoxan; T: taxotere; H: herceptin; CW: chest wall; Sclav: supraclavicular; IM: internal mammary; PAB: posterior axillary boost; AAB: anterior axillary boost.

**Table 2:** Acute locoregional toxicity of concurrent Pazopanib + RT versus RT alone scored using CTCAE.

Locoregional toxicity	Pazopanib + RT		RT Alone	
	Grade 1-2	Grade 3–5	Grade 1-2	Grade 3–5
	*n* affected (total)	*n* affected (total)	* n* affected (total)	*n *affected (total)
Fatigue	12 (12)	0 (12)	25 (25)	0 (25)
Dermatitis radiation	12 (12)	0 (12)	21 (19)	4 (25)
Skin hyperpigmentation	2 (12)	0 (12)	15 (25)	0 (25)
Skin ulceration	1 (12)	0 (12)	0 (25)	0 (25)
Soft tissue fibrosis	0 (12)	0 (12)	0 (25)	0 (25)
Pneumonitis	0 (12)	0 (12)	0 (25)	0 (25)
Diarrhea	3 (12)	0 (12)	0 (25)	0 (25)
Hypertension	1 (12)	0 (12)	0 (25)	0 (25)
Nausea	2 (12)	0 (12)	0 (25)	0 (25)
Anorexia	1 (12)	1 (12)	0 (25)	0 (25)

RT: radiotherapy; CTCAE: Common Terminology Criteria for Adverse Events (v4.0).
